# Gay- and Lesbian-Sounding Auditory Cues Elicit Stereotyping and Discrimination

**DOI:** 10.1007/s10508-017-0962-0

**Published:** 2017-03-15

**Authors:** Fabio Fasoli, Anne Maass, Maria Paola Paladino, Simone Sulpizio

**Affiliations:** 1 School of Psychology, University of Surrey, Stage Hill Campus, Guildford, GU2 7XH UK; 2 Center for Psychological Research and Social Intervention, Instituto Universitario de Lisboa, Lisbon, Portugal; 3 Department of Developmental Psychology and Socialization, University of Padua, Padua, Italy; 4 Department of Psychology and Cognitive Science, University of Trento, Trento, Italy; 5 Faculty of Psychology, Vita-Salute San Raffaele University, Milan, Italy

**Keywords:** Stereotypes, Discrimination, Sexual orientation, “Gaydar”

## Abstract

The growing body of literature on the recognition of sexual orientation from voice (“auditory gaydar”) is silent on the cognitive and social consequences of having a gay-/lesbian- versus heterosexual-sounding voice. We investigated this issue in four studies (overall *N* = 276), conducted in Italian language, in which heterosexual listeners were exposed to single-sentence voice samples of gay/lesbian and heterosexual speakers. In all four studies, listeners were found to make gender-typical inferences about traits and preferences of heterosexual speakers, but gender-atypical inferences about those of gay or lesbian speakers. Behavioral intention measures showed that listeners considered lesbian and gay speakers as less suitable for a leadership position, and male (but not female) listeners took distance from gay speakers. Together, this research demonstrates that having a gay/lesbian rather than heterosexual-sounding voice has tangible consequences for stereotyping and discrimination.

## Introduction

Sexual orientation is a social category that, differently from many others that are signaled by clear visual features such as skin color, is not ascertained until the person self-discloses. Yet, people categorize individuals as gay or heterosexual on the basis of indirect cues, including their physical appearance, their body language, and their vocal characteristics (Rieger, Linsenmeier, Gygax, Garcia, & Bailey, 2010; Rule, 2017; Shelp, 2002). Whereas people may find it relatively easy to monitor their appearance and body language, voice may be less controllable than other cues (Fasoli, Maass, & Sulpizio, 2016). Although a growing number of studies have investigated how people use voice to infer sexual orientation (Gaudio, 1994; Munson, 2007; Munson, McDonald, DeBoe, & White, 2006; Rieger et al., 2010; Smyth, Jacobs, & Rogers, 2003; Sulpizio et al., 2015), less is known about the role of auditory information in stereotyping, namely the attribution of traits or characteristics to a person on the basis of shared beliefs regarding the social groups they belong to (Locksley, Hepburn, & Ortiz, 1982). The few studies that have analyzed the consequences of inferred sexual orientation have mainly focused on visual rather than vocal information (Knöfler & Imhof, 2007; Lick & Johnson, 2014). The present work aims to extend this literature by investigating how people react to vocal cues of sexual orientation in terms of stereotypical inferences, social avoidance, and discriminatory behaviors.

At a theoretical level, we argue that vocal cues affect stereotyping and discrimination in much the same ways as do visual cues. Paralleling current theorizing on *social vision* (for a recent overview, see Johnson, Lick, & Carpinella, 2015), we believe that there are two pathways through which *social hearing* may affect inferences, judgments, and behaviors toward speakers: a category- and a feature-based process (Blair, Judd, Sadler, & Jenkins, 2002). As happens for visual perception (Blair et al., 2002), on one side auditory cues may trigger a social categorization (gay vs. heterosexual), which in turn activates corresponding stereotypes and behavioral responses. On the other side, it may activate stereotypes directly, in the absence of categorization, only based on people’s vocal features (see Ko, Judd, & Blair, 2006; Ko, Judd, & Stapel, 2009). Concretely, speakers may be stereotyped and/or discriminated either because they are categorized as gay (category-based process) or simply because they sound feminine (feature-based process). In our work, we address these issues by investigating how listeners react to vocal cues when no explicit mention of sexual orientation is made.

### Detection of Sexual Orientation

The above distinction between category- and feature-based processes is important, as research on categorization of sexual orientation has provided mixed results with regard to people’s ability to detect sexual identity (so called “gaydar”). Studies have shown that people are quite accurate in judging whether a person is gay or heterosexual on the basis of nonverbal behavior, including mannerisms, gestures, and gait (Ambady, Hallahan, & Conner, 1999). Observers also seem, to some degree, able to guess a person’s sexual orientation from facial features even when peripheral cues such as hairstyle are removed (e.g., Rule & Ambady, 2008, 2009; Tskhay, Feriozzo, & Rule, 2013).

Studies conducted on voice, mostly done in English, have at times provided evidence that people are accurate in detecting sexual orientation from vocal cues (Gaudio, 1994; Rieger et al., 2010; Valentova & Havlíček, 2013). However, other studies have often disproven this claim and suggested that people distinguish between gay and heterosexual individuals on the basis of the stereotypical idea of how gays appear or sound (Smyth et al., 2003; Sulpizio et al., 2015). According to gender inversion theory (Kite & Deaux, 1987), gay/lesbian individuals are expected to be similar, even in terms of face and voice, to opposite-sex heterosexuals. This assumption of gender-atypicality may in part reflect actual differences in personality (Lippa, 2005, 2008). Different lines of research concur that observers tend to rely on gender-typicality versus gender-atypicality when categorizing and drawing conclusions about a person’s sexual orientation, independent of the actual diagnosticity of such cues (Cox, Devine, Bischmann, & Hyde, 2016; Freeman, Johnson, Ambady, & Rule, 2010; Lick & Johnson, 2014; Lyons, Lynch, Brewer, & Bruno, 2014; Rule, Johnson, & Freeman, 2016; see also D’Augelli, Grossman, & Starks, 2008; Dunne, Bailey, Kirk, & Martin, 2000). Also, listeners’ voice-based categorization often reflects their expectations of how heterosexual (vs. gay) people are likely to speak. Listeners make reliable and consensually shared distinctions between gay- and heterosexual-sounding speech that do not necessarily correspond with the speakers’ self-definition (Smyth et al., 2003; Sulpizio et al., 2015; Zimman, 2010). Interestingly, the degree to which people use gender-stereotypical or gender-atypical cues (female features in men and male features in women) to infer sexual orientation depends greatly on people’s beliefs of how diagnostic such cues are (Cox et al., 2016; Freeman et al., 2010; Lick & Johnson, 2014; Stern, West, Jost, & Rule, 2013).

Together, these lines of research suggest that people rely heavily on gender-atypicality (vs. typicality) when judging the sexual orientation of others. However, the fact that the categorization as gay or lesbian is driven by gender-atypical features does not exclude the possibility that gender-atypical features may also affect social perception in the absence of categorization. Extrapolating from Blair et al.’s (2002) and Johnson et al.’s (2015) reasoning, we hypothesize that (vocal) gender-atypicality will affect reactions toward the speaker either via categorization or via directly. We argue here that both categorization of sexual orientation and the perception of speech as gender-atypical (independent of categorization) may emerge spontaneously and lead to similar outcomes that will be discussed below.

### Consequences of Perceiving Sexual Orientation from Voice

Although prior research has largely been mute on this issue, there is reason to believe that having a gay- or lesbian-sounding voice may have consequences for how the speaker is perceived, evaluated, and treated by others. Knöfler and Imhof (2007) have shown that, although unaware of the partner’s sexual orientation, heterosexuals showed different nonverbal behaviors when interacting with a gay (vs. a heterosexual) same-sex partner. In particular, interactions between a gay and a heterosexual person led to more self-touch, fewer and shorter eye gazes, and a reduced preference for a full face-to-face communication. Moreover, research on voice (unrelated to sexual orientation) has shown that vocal information affects the inferences listeners make about speakers’ personality (Aronovitch, 1974; McAleer, Todorov, & Belin, 2014; Scherer, 1978), suggesting that voice is a relevant cue in everyday interactions. However, to our knowledge, only one study has addressed the question of whether being exposed to a gay-sounding voice elicits discrimination. Gowen and Britt (2006) tested the interplay between vocal and explicit information about an individual’s sexual orientation on stigmatizing reactions. They found that voice itself did not affect discrimination of the target, unless it violated expectations (e.g., a heterosexual man with a gay voice). Although interesting, these findings provide only initial evidence for the relation between vocal cues and stigmatization.

Our work extends this line of research by, first, investigating how voice affects gender stereotyping, social distance, and behavioral intentions when no explicit information about the speaker’s sexual orientation was available. Thus, it simulates everyday situations in which listeners are not aware of speakers’ actual sexual orientation. In particular, we refer to stereotyping as the process of attributing gender-atypical traits, characteristics, and interests to a person based on the fact that gay men are usually associated with femininity and lesbian women with masculinity (Blashill & Powlishta, 2009; Kite & Deaux, 1987). Hence, in this work, stereotyping refers to an attributional process irrespective of whether these associations reflect actual differences between gays/lesbians and heterosexuals (see Devine, 1989; Judd & Park, 1993).

Second, our studies investigated discriminatory behaviors that may emerge in a work-related context and specifically in the hiring process. Discrimination of gay/lesbian individuals at work and in hiring process is common (Ahmed, Andersson, & Hammarstedt, 2013; Badgett, Lau, Sears, & Ho, 2007; Patacchini, Ragusa, & Zenou, 2014), and even indirect cues such as being involved in a LGBT association decreases chances to get appointed for a job (Tilcsik, 2011). The fact that sexual orientation is rarely mentioned explicitly in job interviews makes investigating the effects of nonverbal information, including voice and appearance, all the more relevant. Although the role of appearance in job interviews is well known (Atkins & Kent, 1988; Juodvalkis, Grefer, Hogue, Svyantek, & DeLamarter, 2003), it remains unclear whether a gay-/lesbian-sounding voice influences the hiring process and whether it does so specifically for high-status masculine jobs that require leadership abilities. We will therefore test here whether gay-/lesbian (vs. heterosexual)-sounding candidates will be discriminated in simulated hiring decisions when applying for upper management positions typically associated with masculine traits.

Third, this research addressed whether any observed effect was due to category- and/or feature-based process of speakers and hence contributes to the understanding of what drives stereotyping and discrimination. Fourth, different from Gowen and Britt’s (2006) study that focused only on men, we will consider both male and female speakers, thus overcoming a common male bias in research on gaydar. Finally, in the last study we will directly compare the impact of auditory and visual cues of sexual orientation.

### Overview

We investigated whether voice influences the attribution of stereotypical traits, sports, and fields of study, and the intention to interact with the speaker (Study 1a and 1b). In the subsequent studies (2a and 2b), we focused on the role of voice in the hiring process for a stereotypically masculine job for which leadership abilities were required.

In both sets of studies, we investigated two interrelated questions, one of more applied, the other of more theoretical interest. On the one hand, we tested whether voice would lead to stereotypical inferences and discrimination. On the other hand, we investigated whether voice-based stereotyping and discrimination were driven by categorization or by voice features, or whether both (category- and feature-based) processes contributed independently.

Different from previous studies on gay voice, we were not interested in recognition of sexual orientation per se, but rather in the inferences people draw when they encounter voices that sound gay versus heterosexual. Therefore, rather than selecting larger samples of voices that are more or less representative of the gay or heterosexual population (as is generally done in studies on “gaydar”), we opted for a different research strategy by purposefully selecting, on the basis of prior research, a small sample of voices that had a relatively high likelihood of being perceived as gay versus heterosexual. Thus, our interest concerns how people react to “prototypical” gay or heterosexual voices, without making any claims about the representativeness of these voices.

### Study 1: Inferring Interests and Traits from Voice

In Study 1, we examined whether heterosexual listeners made stereotypical attributions in line with the speakers’ perceived sexual orientation. Participants listened to the voices of two speakers who pronounced a single sentence of neutral content, unrelated to sexual orientation, and then evaluated the speakers’ likely personal interests (i.e., sports and fields of study) and personality traits. Importantly, sexual orientation of the speaker was never explicitly mentioned. As gender inversion theory (Kite & Deaux, 1987) suggests that gay/lesbian individuals are perceived similar to opposite-sex heterosexuals, we hypothesized that listeners would attribute more feminine (and fewer masculine) sports, fields of study, and traits to gay than to straight male speakers (Study 1a). The opposite prediction (more masculine and less feminine attributions) was advanced for lesbian compared to straight female speakers (Study 1b).

In addition, we tested heterosexuals’ behavioral intentions by asking participants to choose one of two speakers as an interaction partner for a subsequent discussion. We expected that gay and lesbian speakers would be chosen less frequently than their heterosexual counterparts and that this bias would be particularly pronounced for gay men, given that homophobia is generally stronger toward gay males than toward lesbians (Kilianski, 2003).

## Method

### Participants

Participants were recruited online through students’ contacts at a large Italian university. In Study 1a, the final sample consisted of 81 participants (37 males, *M*_age_ = 21.89 years, SD = 3.73). This sample was obtained after excluding participants who self-identified as non-heterosexuals (*n* = 9) or who had reported technical problems (*n* = 5) from the 95 participants who had completed the survey.

In Study 1b, after excluding participants who had technical problems (*n* = 2) and self-identified as non-heterosexual (*n* = 12), the final sample consisted of 40 participants (14 males, *M*_age_ = 23.28 years, SD = 3.67).

### Measures and Procedure

In Study 1a, four male speakers were selected from a database by Sulpizio et al. (2015) whose speakers had been rated for sexual orientation on a scale from 1 (completely homosexual) to 6 (completely heterosexual) and whose vocal cues were analyzed. Of the 20 speakers of Sulpizio et al.’s study, we chose the two speakers who had the highest likelihood to be perceived as gay (*M* = 1.49, SD = .63) and two speakers who were consistently perceived as heterosexual (*M* = 4.60, SD = .93), *t*(85) = −27.62, *p* < .001, *d* = 5.99. The way in which their voice was perceived also corresponded to the speakers’ self-identified sexual orientation.

The same procedure was applied in Study 1b, except for the fact that female rather than male voices were selected. Speakers were chosen on the basis of a pretest (*n* = 62) where 9 female speakers’ sexual orientation was rated on a scale from 1 (completely heterosexual) to 6 (completely lesbian). The voices of the two lesbian (*M* = 2.71, SD = .91) and the two heterosexual speakers (*M* = 4.57, SD = 1.12), selected for this study, were perceived as significantly different in terms of sexual orientation, *t*(61) = −10.06, *p* < .001, *d* = 2.58.

Thus, in both studies speakers not only were self-identified as either gay/lesbian or heterosexual, but their voices had a relatively high probability to be distinguished. Each speaker pronounced the exact same neutral sentence (i.e., “il cane correva nel parco/the dog ran in the park”).^1^

Participants in both studies were recruited via email or social networks and provided with a link to the online survey. They were informed that we were interested in how people form impressions about individuals on the basis of their voice and that they would listen to short audio files. Participants were invited to turn on the volume of the computer speakers and to disconnect any device that may produce noise (cell phone, Skype, etc.). Every participant listened to only one gay/lesbian and one heterosexual speaker among the four speakers selected for these experiments. The pair of speakers and the order of speakers’ presentation were counterbalanced across participants.

After listening to each speaker, participants estimated the likelihood that the speaker played different sports and was enrolled in different fields of study. Participants were provided with a list of three typically male (football, rugby, and cycling) and three typically female sports (dance, aerobic, and volleyball), and with a list of three masculine (engineering, physics, biology) and three feminine fields of study (psychology, education, foreign languages).^2^ Participants indicated the likelihood that the speaker was engaged in each sport or field of study on a scale from 1 (not at all likely) to 5 (very likely). Then, participants rated the speaker on a list of 10 feminine (i.e., caring, emotional, tidy, creative, romantic, sensitive, insecure, effeminate, mischievous, and gossipy) and on 10 masculine traits (i.e., dominant, vigorous, leader, determined, practical, aggressive, rude, violent, arrogant, and overbearing).

Finally, behavioral intention was measured. Participants were asked to imagine being part of a workshop on “social networks and new generations.” They were told this workshop was supposed to start with a discussion between two people. They were asked to choose, as a partner for the discussion, one of the two speakers they had listened to before. After making their choice, they responded to three items regarding the interaction partner (i.e., “How much would you like to know your partner’s opinion about the workshop topic?”, “How friendly do you perceive the person you have chosen?”, and “Would you like to get to know this person better and meet him [her] in another context [e.g., bar]?”). Given the good internal reliability of these three items (*α* = .64 for Study 1a and *α* = .82 for Study 1b), they were averaged. However, when comparing the judgments of those who chose the gay-sounding and those who chose the straight-sounding speaker, we found no significant difference either for the male or for the female voice. Hence, this variable will not be discussed further.

Lastly, participants completed either the Attitude Toward Gay Men Scale (ATG; Study 1a) or the Attitude Toward Lesbians scale (ATL; Study 1b) (Herek, 1998), depending on the speakers’ gender. Both were assessed on 5-point scales (from 1 = completely disagree to 5 = completely agree) and were internally reliable (*α* = .89 for ATG and *α* = .71 for ATL). Only at that point participants were asked to guess the sexual orientation of each speaker by selecting one out of three options (i.e., homosexual, bisexual, and heterosexual) and reported their demographic information (i.e., age, gender, and sexual orientation). Finally, participants read a debriefing about the aim of the study and were thanked for their participation.

## Results

### Study 1a: Male Targets

Gay speakers were identified as gay or bisexual by 75% of the participants, and heterosexual speakers were identified as such by 89% of the participants. Also, 67% of the participants were able to correctly recognize both speakers and only one participant got both wrong.

#### Sports, Field of Study, and Traits

For each of the three domains (sports, fields of study, and traits), masculine and feminine items were averaged separately (for alphas, see Table 1). We then conducted a 2 (Participant Gender) × 2 (Typicality: Masculine vs. Feminine) × 2 (Speaker’s Sexual Orientation: Gay vs. Heterosexual) repeated-measures ANOVA with the last two variables as within-subjects factors. In each case, we found a significant interaction between Typicality and Speaker’s Sexual Orientation, *F*(1, 79) = 160.99, *p* < .001, *η*_*p*_^2^ = .67 for Sports, *F*(1, 79) = 35.74, *p* < .001, *η*_*p*_^2^ = .3 for Fields of Study, and *F*(1, 80) = 154.23, *p* < .001, *η*_*p*_^2^ = .66 for Traits. For all three domains, we found that participants attributed more feminine items to the gay than to the heterosexual speakers, but more masculine items to the heterosexual than to the gay speakers (Table 1).

**Table 1:** Means (SD) of feminine and masculine items attributed to male speakers (Study 1a)

	Alpha	Gay speakers	Straight speakers
Sports			
Feminine	*α* = .75	2.58 (.90)^a^	1.89 (.71)^b^
Masculine	*α* = .71	1.94 (.74)^b^	2.84 (.91)^a^
Field of study			
Feminine	*α* = .72	3.11 (.89)^c^	2.44 (.84)^d^
Masculine	*α* = .69	2.34 (.86)^d^	2.91 (.95)^c^
Traits			
Feminine	*α* = .83	3.13 (.61)^e^	2.24 (.54)^f^
Masculine	*α* = .82	1.72 (.48)^g^	2.27 (.58)^f^

Ratings ranged from 1 (not at all likely) to 5 (very likely). Means comparing gay and straight speakers that do not share the same subscript within each domain were significantly different from each other according to Bonferroni multiple comparisons

Sport: *p* < .001; field of Study: *p* < .002; traits: *p* < .001

Only in the case of sports, we found an effect of Participant Gender on attribution of masculine and feminine sports to gay and heterosexual speakers, *F*(1, 79) = 5.51, *p* = .02, *η*_*p*_^2^ = .06. In particular, as shown by pairwise comparisons, whereas masculine sports were equally attributed to gay speakers by male (*M* = 1.86, SD = .61) and female participants (*M* = 2.01, SD = .84; *p* = .36), male participants (*M* = 2.84, SD = .93) attributed more feminine sports to gay speakers than female participants (*M* = 2.35, SD = .82; *p* = .02) did. No significant gender differences were found in attribution of masculine/feminine sports to heterosexual speakers (*p*s > .30).^3^

#### Behavioral Intentions

The majority (69%) of participants chose the heterosexual speaker as a potential interaction partner, whereas only 31% of participants chose the gay speaker. These choices differed significantly from chance level, *χ* ^2^ = 22.22, *p* < .001, and revealed that this was true only for male but not for female participants, *χ* ^2^ = 4.55, *p* = .03. Male participants showed a strong preference for the heterosexual speaker who was chosen 81% of the time. In contrast, female participants did not show a significant difference for the gay (41% of participants) versus the heterosexual speaker (59% of participants).

#### Correlation Analyses

To test whether attitudes toward gay men (ATG) (overall *M* = 2.16, SD = .85) would be associated with the degree of stereotyping and the choice of the interaction partner, we first calculated a Stereotyping Index for each characteristic (sport, field of studies, traits) by summing the attribution of gender-typical (minus atypical) characteristics to heterosexual and of gender-atypical (minus typical) characteristics to the gay/lesbian speaker. The greater the value, the more heterosexual speakers were associated with gender-typical and gay speakers with atypical characteristics. ATG did not correlate with the stereotyping indices (*r*s < .18) nor was it predictive of the choice of the interaction partner (*r* = .10). It also did not correlate when considering the type of speaker, the type of attribution (masculine vs. feminine), and the domain (sport, field of studies, and traits) separately (*r*s < .17).

### Study 1b: Female Targets

The heterosexual speaker was identified correctly by 82% of the participants, whereas the lesbian speaker was identified correctly (as either lesbian or bisexual) only by 54% of the participants. Overall, approximately half of the participants (49%) identified both speakers correctly and five participants (13%) got both wrong.

#### Sports, Field of Study, and Traits

As in Study 1a, ratings on masculine and feminine items of the three domains were averaged and submitted to a 2 (Participant gender) × 2 (Typicality: Masculine vs. Feminine) × 2 (Speaker’s Sexual Orientation: Lesbian vs. Heterosexual) repeated-measures ANOVA with the last two variables as within-subjects factors. A significant interaction between Typicality and Speaker’s sexual orientation was found for Sport, *F*(1, 33) = 28.78, *p* < .001, *η*_*p*_^2^ = .46, for Field of Study, *F*(1, 33) = 18.62, *p* < .001, *η*_*p*_^2^ = .36, and for Traits, *F*(1, 34) = 40.98, *p* < .001, *η*_*p*_^2^ = .55. Participants attributed more masculine items to the lesbian than to the heterosexual speakers, whereas the opposite was true for the feminine items (see Table 2).^4^

**Table 2:** Means (SD) of feminine and masculine items attributed to female speakers (Study 1b)

	Alpha	Lesbian speakers	Straight speakers
Sports			
Feminine	*α* = .64	1.98 (.73)^a^	3.21 (1.05)^b^
Masculine	*α* = .65	2.37 (1.05)^a^	1.54 (.67)^c^
Field of study			
Feminine	*α* = .59	2.42 (.89)^d^	3.29 (1.12)^e^
Masculine	*α* = .75	2.39 (1.12)^d^	1.76 (.73)^f^
Traits			
Feminine	*α* = .73	2.12 (.45)^g^	2.24 (.54)^g^
Masculine	*α* = .92	2.41 (.86)^g^	1.79 (.69)^j^

Ratings ranged from 1 (not at all likely) to 5 (very likely). Means comparing lesbian and straight speakers that do not share the same subscript within each domain were significantly different from each other according to Bonferroni multiple comparisons

Sport: *p* < .001; field of study: *p* < .003; traits: *p* < .001

#### Behavioral Intentions

Participants were approximately equally likely to select the lesbian (46.2%) and the heterosexual speaker (53.8%), *χ* ^2^ < 1, and no significant gender differences emerged.

#### Correlational Analyses

Again, as in Study 1a, we calculated a Stereotyping Index for sports, fields of studies, and traits and then correlated the ATL (*M* = 1.97, SD = .56) with these indices. ATL did not predict stereotyping nor was it predictive of partner choice (*r*s < .26).

### Gay/Lesbian Categorization or Gender-Atypical Vocal Features?

The results of both studies clearly showed that gender-atypical characteristics were attributed more to gay and lesbian than to heterosexual speakers. However, this result is open to two different explanations, one based on social categorization, the other on a direct effect of voice on stereotyping (Blair et al., 2002; Johnson et al., 2015). People may sense that the minority speaker is gay/lesbian and therefore attribute those characteristics to the speaker that are typical of the opposite sex (Kite & Deaux, 1987; Lippa, 2008). Alternatively, sexual orientation categorization may play no role at all, but perceivers may simply find the male voice less masculine and the female voice less feminine and therefore attribute corresponding interests and traits to that person, without inferring that the person was gay or lesbian. In an attempt to distinguish the two explanations, we compared those participants who had correctly identified the sexual orientation of both speakers with those who did not. If the gender-atypicality of the voice was the driving force, listeners should make gender-inverted inferences regardless of whether they did or did not correctly report the sexual orientation of the speaker. In this case, stereotyping and discrimination are presumably feature-based (Blair et al., 2002). In contrast, if social categorization was the driving force, then gender-inverted inferences should only be shown by those who correctly identified the speakers as heterosexual or gay/lesbian, respectively. Of course, it is also possible that both processes operate, in which case vocal features should be sufficient to induce (above chance) stereotyping and discrimination even in the absence of social categorization, but correct categorization of speakers as gay/lesbian versus heterosexual may enhance these effects above and beyond the feature-based process.

**Fig. 1:**
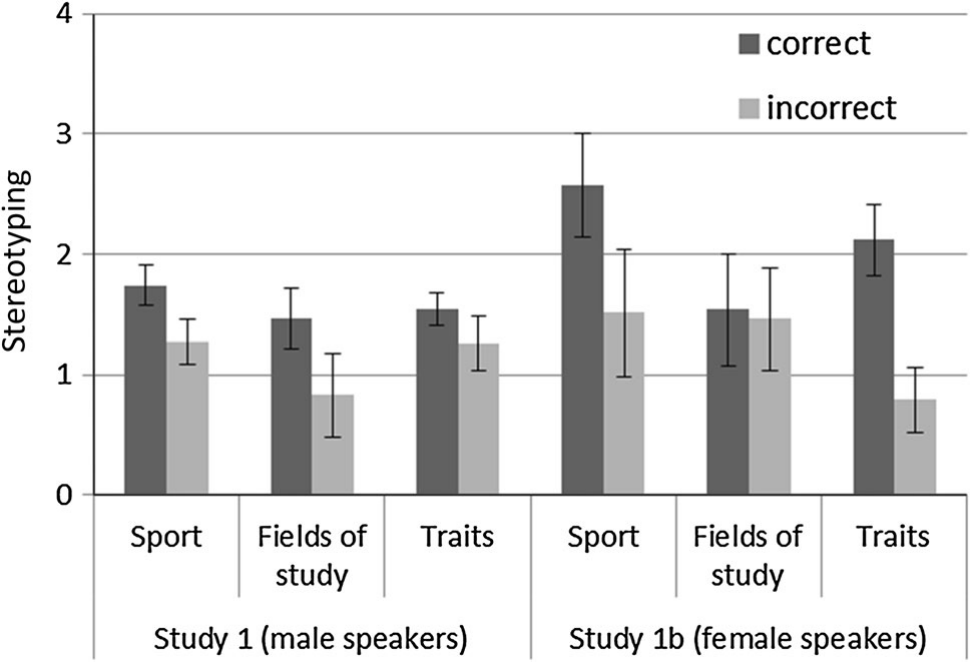
Relative attribution of stereotypically male versus female characteristics to gay/lesbian versus straight voices among participants who did or did not correctly identify the speakers’ sexual orientation (Study 1a and Study 1b)

To simplify interpretation, we used the Stereotyping Indices as dependent variables, with greater values, indicating that the heterosexual speakers were associated more with gender-typical and the gay/lesbian speakers with atypical characteristics. A 2 (Accuracy: Correct vs. Incorrect Identification of Speaker) × 3 (Domain: Sports, Field of Studies, Traits) ANOVA with repeated measures on the latter variable revealed a main effect of Accuracy such that those who recognized the sexual orientation of the speakers correctly engaged in stronger stereotyping than those who did not, for male (Study 1a), *F*(1, 79) = 4.58, *p* = .03, *η*_*p*_^2^ = .05, and, albeit short of significance, for female speakers (Study 1b), *F*(1, 33) = 3.59, *p* = .06, *η*_*p*_^2^ = .10. Thus, speakers were stereotyped more strongly when they were also categorized as heterosexual versus gay/lesbian. However, in all cases, regardless of correct categorization, means differed significantly from 0 (one-sample *t* tests, *p*s < .05), suggesting a general tendency to associate heterosexual voices with gender-typical and gay/lesbian voices with atypical characteristics (see Fig. 1). Together, this pattern suggests that gay/lesbian speakers may be stereotyped either because their voices sound gender-atypical or because they are perceived as gay/lesbian, providing support to the dual-path model (Blair et al., 2002; Johnson et al., 2015).

A similar, but weaker, pattern emerged for the choice of the male interaction partner in Study 1a. Those who did not identify the sexual orientation of the speakers had a small but nonsignificant preference for the heterosexual male (63%, binomial test, n.s.), whereas those who had correctly identified the speakers’ sexual orientation showed a clear preference for the heterosexual speaker (72%, binomial test, *p* < .001). For women (Study 1b), there was no systematic association between recognition of sexual orientation and choice of interaction partner.

The question whether sexual orientation-based categorization contributed above and beyond the effects of gender-atypical sound can also be addressed from a different vantage point. One may completely ignore the accuracy of the categorization but simply ask whether the two speakers were perceived as being of the same or of different sexual orientation (regardless of accuracy of this categorization). Note, however, that the large majority of participants who categorized the speakers as of different sexual orientation also classified them correctly (79% in the case of female and 98% in the case of male voices). If categorization plays an important role (as part of the indirect path), then participants who perceived the two speakers as being of different sexual orientation should show greater stereotyping. We therefore conducted 2 (Speakers’ Differentiation: Speakers perceived of the Same vs. Different Sexual Orientation) × 3 (Domain: Sports, Field of Studies, Traits) ANOVAs for both male and female voices. A significant main effect of speakers’ differentiation suggests that participants who categorized the two male speakers as being of different sexual orientation (*M* = 1.59, SD = .93) showed greater stereotyping than those who categorized them the same (*M* = 1.08, SD = .89), *F*(1, 79) = 5.54, *p* = .021, *η*_*p*_^2^ = .07. Similarly, for female voices, those who categorized the two speakers as belonging to distinct sexual orientation categories showed greater stereotyping (*M* = 2.12, SD = 1.25) than those who categorized them in the same way, that is, both as gay or both as heterosexual (*M* = .83, SD = 1.08), *F*(1, 33) = 9.01, *p* = .005, *η*_*p*_^2^ = .21. This suggests that sexual orientation categorization (whether right or wrong) increased stereotyping. However, again, for both male and female voices, the stereotyping index differed reliably from 0 (one-sample *t* tests, *p* < .001 for male voices and *p* < .03 for female voices), even in the absence of categorization, suggesting that gender-atypical sound produced stereotyping even in the absence of a categorical distinction between the two speakers. This again supports the view that categorization is not necessary for stereotyping to occur and that a feature-based process operates in addition to the more intuitive category-based process.

## Discussion

Study 1 provided evidence that listeners used vocal cues to make inferences about the speakers and that these inferences were stereotype-based and gender-inverted (Kite & Deaux, 1987; see also Lippa, 2008, for actual differences in occupational preferences and in masculinity–femininity). On the one hand, the gay speakers were associated with more feminine and fewer masculine characteristics in three different domains. On the other hand, lesbian speakers were more likely to be associated with masculine than to feminine characteristics. Vocal information that cues gayness induced listeners to deny the speakers those qualities that are considered typical of their gender and to over-attribute those qualities that are typical of the opposite gender. Interestingly, this happened despite the fact that no mention was made of sexual orientation and even when listeners encountered difficulties in categorizing the gay or lesbian speakers as homosexuals.

Does this mean that sexual orientation plays no role at all? Our supplementary analyses suggest otherwise: Perceived masculinity–femininity alone was sufficient to produce an inversion effect, but this effect became stronger when inferred sexual orientation came into play. Thus, counter-stereotypical traits and preferences are attributed to atypical-sounding men and women, even when they are perceived as heterosexual, but this occurs to a larger extent when they are also perceived as gay or lesbian, respectively. These results demonstrate that the mere sound of voice is sufficient to trigger stereotyping, but that social categorization adds to stereotyping above and beyond the direct effect of voice on stereotyping. Hence, *social hearing* has a strong impact on those who are listening, but it may achieve its effect through two distinct routes as hypothesized by Johnson et al. (2015) for social vision.

Our findings also provide initial evidence that voice influences behavior intentions. Indeed, male participants were more likely to avoid male gay speakers, particularly when they had correctly identified the speaker as gay. This explanation is in line with research, showing that heterosexual men distance themselves from gay men or individuals deviating from masculine gender norms (Falomir-Pichastor & Mugny, 2009), as well as with those demonstrating that men have a more negative attitude toward gay men than women do (Herek, 2000; Kilianski, 2003; Kite & Whitley, 1996; also found in our own data where women reported lower ATG than men, *t*[79] = 4.02, *p* < .001). However, our results are also open to a different explanation. The preference for the heterosexual partner may simply reflect the wish to discover the individual’s sexual orientation and/or to interact with the (heterosexual-sounding) man who may represent a potential in-group member.

### Study 2: Inferring Leadership Abilities from Voice

Leadership is defined as “a process of social influence in which one person is able to enlist the aid and support of others in the accomplishment of a common task” (Chemers, 2000, p. 27). However, in common sense, the leader is a person who possesses traits such as dominance, assertiveness, and intelligence, that is, traits that are believed to be more typical of men than women, a phenomenon known as “think-manager-think-male” effect (see Koenig, Eagly, Mitchell, & Ristikari, 2011; Powell, Butterfield, & Parent, 2002; Schriesheim, Castro, & Cogliser, 1999; Sczesny, 2003). Hence, when an individual is perceived as incongruent with this typically masculine role, s/he is discriminated against (Garcia-Retamero & Lopez-Zafra, 2006; Rojahn & Willemsen, 1994). This is the case of women in higher managerial and leadership positions (Eagly & Karau, 2002) who may represent a threat to male dominance (Rudman, Moss-Racusin, Phelan, & Nauts, 2012).

In modern Western societies, LGBT individuals can be successful even if openly homosexual (see the “Who’s Who: Top 50 OUTstanding in Business List,” 2013). Fassinger, Shullman, and Stevenson (2010) suggested that LGBT individuals may also be good leaders because, among other reasons, they have developed good communication and coping skills by facing prejudice (see also Eagly & Chin, 2010; Snyder, 2006). At the same time, gay/lesbian leaders may have to deal with different sources of prejudice and stereotyping. Indeed, gay men and lesbian women often encounter career barriers that are related to gender roles (Parnell, Lease, & Green, 2012). Gay men may be perceived as lacking those masculine characteristics that leaders should have. Conversely, lesbian women may be seen as deviating from typical gender roles and as a threat to male dominance, thereby becoming target of double discrimination, as women and as lesbians (Fassinger et al., 2010).

“Diversity” of leaders, including sexual orientation, continues to be an under-investigated issue (Eagly & Chin, 2010). Whether sexual orientation matters in leadership and how auditory and visual cues affect leaders’ evaluation remains an underexplored issue, yet there is indirect evidence that links dominance and leadership perception to vocal and facial features. Prior research has shown that visual and vocal cues influence hiring decision (Atkins & Kent, 1988; Juodvalkis et al., 2003; Petersen & Togstad, 2006) and that masculine facial and vocal features are associated with dominance and power (Boothroyd, Jones, Burt, & Perrett, 2007; Fink, Neave, & Seydel, 2007; Feinberg et al., 2006; Jones, Feinberg, DeBruine, Little, & Vukovic, 2010). Facial cues and nonverbal behaviors (shown in short videos) have also been found to relate to leadership perception and success (Rule & Ambady, 2011; Rule, Ishii, & Ambady, 2011; Tskhay, Xu, & Rule, 2014). Similarly, voice features (e.g., pitch and formant frequencies) influence dominance attribution and leadership perception (Klofstad, Anderson, & Peters, 2012; Puts, Hodges, Cardenas, & Gaulin, 2007; Tigue, Borak, O’Connor, Schandl, & Feinberg, 2012). Nevertheless, all of these studies focused on masculinity (vs. femininity) but did not test whether sexual orientation conveyed by auditory or visual features affects perceived leadership abilities in similar ways.

In Study 2, we examined whether having a gay-/lesbian-*sounding voice* or a gay-/lesbian-*looking face* induced discriminatory behaviors toward an applicant for a leadership position. In particular, we tested whether listeners and observers evaluated candidates differently, depending on the vocal or facial features indicative of different sexual orientation. For male candidates, we hypothesized that a gay-sounding voice and a gay-looking face would lead to a more negative evaluation of the candidate, including a reduced willingness to hire him and the attribution of a lower salary. For female candidates, the same prediction was advanced. Indeed, as suggested by Fassinger et al. (2010), candidates perceived as lesbian are expected to be subject to double discrimination, as women and as lesbian, and hence be discriminated even more than heterosexual women.

## Method

### Participants

In Study 2a, participants were recruited through students’ contacts using a snowball procedure and provided with the link to an online survey. The final sample consisted of 63 participants (35 males and 28 females; *M*_age_ = 26.57 years, SD = 5.59). From the initial sample of 117 participants who had access to the survey, we excluded those who identified as non-heterosexual (*n* = 9), reported technical problems (*n* = 13), failed to complete the survey (*n* = 15), or did not correctly recall the job vacancy (*n* = 17).

In Study 2b, groups of students were tested simultaneously in the same room. Each participant individually completed an online survey using headphones. The final sample consisted of 92 participants (3 males and 89 females, *M*_age_ = 19.45 years, SD = 1.39). From the initial sample of 152 participants, we excluded those who identified as non-heterosexual (*n* = 8), reported technical problems (*n* = 15), or failed to correctly recall the job vacancy (*n* = 23).

Notice that analyses performed including those who did not correctly remember the target job showed the same pattern of results in both studies.

### Measures and Procedure

Speakers were the same as those in Study 1a and 1b. However, in Study 2a, male speakers pronounced a different sentence, namely “Mi chiamo Luca e ho 32 anni, vengo da Vicenza” (“My name is Luca and I am 32, I come from Vicenza”). Female speakers in Study 2b pronounced the following sentence: “Vengo da Verona e mi chiamo Giulia” (I come from Verona and my name is Giulia).^5^

For Study 2a, 26 pictures, half of gay and half of heterosexual male faces, were selected from the TriesteDataBase (Piccoli, Carnaghi, & Foroni, 2015) and pretested with regard to sexual orientation. Background and other elements (e.g., hair) were removed, leaving only the facial features, including eyes, nose, and mouth. Pretest participants (*N* = 14) saw one picture at a time and indicated the sexual orientation of each target on a scale from 1 (exclusively heterosexual) to 7 (exclusively homosexual). Pictures of two gay-looking (*M* = 4.68, SD = .69) and two heterosexual-looking faces (*M* = 2.14, SD = .57) were selected. These two groups of pictures were significantly different in terms of perceived sexual orientation, *t*(13) = 12.30, *p* < .001, *d* = 6.82. Perceived and actual sexual orientation of the person portrayed in the picture matched. Facial stimuli were presented in combination with the exact information (name, age, city) reported in the speakers’ audio file, except that they were presented in written form.

For Study 2b, 46 pictures of lesbian and straight female faces were collected (following Rule and Ambady’s [2008] procedure) and pretested. Pretest participants (*N* = 11) rated the sexual orientation of each person portrayed in the picture on a scale from 1 (exclusively heterosexual) to 7 (exclusively homosexual). Two lesbian-looking (*M* = 5.23, SD = 1.21) and two heterosexual-looking faces (*M* = 2.27, SD = 1.27; pairwise *t* test: *t*[10] = 4.89, *p* = .001, *d* = 3.09) were selected and perceived sexual orientation matched the sexual orientation of the speaker. Again, each photograph was accompanied by written information about the candidate (name and city) that was identical to the information contained in the audio file.

Participants were told that we were interested in examining how people form impressions about applicants in job interviews. Participants were asked to assume the perspective of an HR manager and to read the job ad that referred to a managerial position in a large company. In particular, they were informed that the company was looking for a CEO for a limited company with good leadership and management skills, global vision of internal processes, and organization of available resources. Next, they were asked to form an impression of the applicant on the basis of limited information such as his/her face or voice. Participants were warned explicitly that it may be difficult to draw inferences from such limited information but that they should try anyway to form an impression. Depending on the experimental condition, participants were either exposed to a visual or auditory stimulus. Participants were informed that the pictures had been cropped with the purpose of testing the impact of limited facial information excluding visual details (e.g., hair, ears, and background).

Participants were then asked to evaluate the applicant’s hireability by answering five items (i.e., “I would entrust the management of the company to this candidate,” “I feel certain about hiring this candidate,” “I consider the applicant suitable for this position,” “I believe that the applicant has the necessary skills to be a good leader,” “In my opinion, the applicant will advance in his career”). Answers were provided on a 5-point scale from 1 (not at all) to 5 (very much). In addition, participants reported the amount of monthly salary they considered adequate (on a scale from 1 = less than 4000 Euro to 7 = more than 7000 Euro). As in Study 1, participants then rated the applicant on 10 feminine and 10 masculine traits (Stereotypicality*)* and completed the ATG/ATL scale. Finally, they indicated the likely sexual orientation of the applicant by choosing between heterosexual, bisexual, and homosexual, and reported in an open-ended question the job the applicant had applied for. At the very end, before providing demographic information (i.e., age, gender, and sexual orientation) and being debriefed, participants indicated whether they had encountered any technical problem with the experimental stimuli.

## Results

### Study 2a: Male Candidate

Overall, sexual orientation of the heterosexual applicant was correctly recognized by the majority (77% of participants), whereas the gay candidate was identified as gay or bisexual by 54% of participants. Across conditions, the heterosexual applicant was correctly recognized both in the visual (77%) and in the auditory conditions (76%). The gay applicant was categorized as homosexual or bisexual by 59% of the participants in the auditory condition and by 50% in the visual condition.

#### Applicant’s Hireability

The scale showed a good internal reliability (*α* = .95) and was scored such that higher scores indicated a more positive evaluation. These ratings were submitted to a 2 (Stimulus: Face vs. Voice) × 2 (Sexual Orientation: Gay vs. Heterosexual) ANOVA where all variables were between-participants factors. A significant interaction between stimulus and sexual orientation, *F*(1, 59) = 14.30, *p* < .001, *η*_*p*_^2^ = .19, was found. Pairwise comparisons (Bonferroni multiple correction) showed no significant difference between the heterosexual (*M* = 2.11 SD = 1.01) and the gay (*M* = 2.21, SD = .59) applicant when he was portrayed through visual cues. In contrast, when judging on the basis of voice, participants evaluated the heterosexual candidate (*M* = 3.63, SD = .76) more positively than the gay candidate (*M* = 2.16, SD = .83; *p* < .001).

#### Salary

The same analysis conducted for the salary recommendation yielded a significant interaction between stimulus and sexual orientation, *F*(1, 59) = 4.89, *p* = .03, *η*_*p*_^2^ = .08. While no significant difference emerged between the heterosexual (*M* = 1.36, SD = .49) and the gay candidate (*M* = 1.31, SD = .63) when their face was shown, in the voice condition the heterosexual candidate (*M* = 2.28, SD = 1.27) was assigned a higher salary than the gay applicant (*M* = 1.27, SD = .45; *p* = .001).

#### Traits

An index of masculine traits (*α* = .90) and one of feminine traits (*α* = .90) was created by averaging participants’ ratings. We then performed a 2 (Stimulus: Face vs. Voice) × 2 (Sexual orientation: Gay vs. Heterosexual) × 2 (Traits: Feminine vs. Masculine) ANOVA where the first two factors were between-subjects variables and the last a within-subjects factor. Analysis yielded a significant interaction between sexual orientation and traits, *F*(1, 58) = 74.50, *p* < .001, *η*_*p*_^2^ = .56. Masculine traits were attributed more to the heterosexual (*M* = 2.93, SD = .71) than to the gay applicant (*M* = 1.87, SD = .49; *p* < .001). In contrast, feminine traits were attributed more to the gay (*M* = 3.26, SD = .62) than to the heterosexual candidate (*M* = 2.25, SD = .57; *p* < .001).^6^

#### Correlation Analyses

Correlational analyses were performed to test the link between ATG (*α* = .89, overall *M* = 2.36, SD = .87) with applicant’s hireability, salary, and gay stereotyping (calculated by subtracting masculine traits from feminine traits). Results showed that ATG did not correlate with the other variables (*r*s < .13, *p*s > .32). However, stereotypes did significantly and negatively correlate with applicant’s hireability, *r*(62) = −.48, *p* < .001, and salary, *r*(62) = −.44, *p* < .001. The more feminine the candidate was perceived, the lower the hireability likelihood and the lower the salary allocated to him.

#### Indirect Effect

The significance of the indirect pathway from applicants’ sexual orientation to hireability via stereotyping was assessed using bias-corrected confidence intervals, calculated using 5000 bootstrap resamples (Preacher & Hayes, 2008). This was done first regardless of the type of stimulus participants were exposed to and then separately for visual and auditory information. Overall, a significant indirect pathway of the applicant’s sexual orientation on hireability, mediated through stereotypes, emerged (*β* = .25, SE = .37; CI_95_ = .07, 1.25). Similarly, in the auditory cue condition, the confidence interval of the indirect effect did not include zero (*β* = −.72, SE = .35; CI_95_ = .35, 1.48), hence confirming this mediating pattern. Instead, in the visual condition, the confidence interval of indirect effect did include zero (*β* = −.48, SE = .51; CI_95_ = −.29, 1.29). Together, these results reflect the fact that gay-sounding voices led to gay stereotyping which, in turn, led to a lower likelihood to hire the applicant.

### Study 2b: Female Candidates

The heterosexual female candidate was correctly perceived as such by 93.4% of participants, whereas the lesbian applicant was perceived as heterosexual by 87.1% of participants. This failure to recognize the sexual orientation of the lesbian applicant was found in both the auditory and the visual conditions: 86.7% of participants perceived the lesbian applicant as heterosexual when listening to the voice, as did 87.5% of participants who saw the photograph. The heterosexual applicant was perceived as such by 90.6% of participants in the voice and by 96.6% in the photograph condition, respectively. Hence, regardless of the stimulus type, the candidate was consistently categorized as heterosexual.

#### Applicant’s Hireability

Again, an index was created by averaging participants’ ratings of the five items (*α* = .85). The higher the score, the more likely the applicant’s hireability. Hireability was then submitted to a 2 (Stimulus: Face vs. Voice) × 2 (Sexual Orientation: Lesbian vs. Heterosexual) ANOVA where all variables were between-participants factors. Results showed a main effect of Stimulus, *F*(1, 87) = 6.49, *p* = .01, *η*_*p*_^2^ = .07. Participants reported more positive evaluations of the applicant when they saw her face (*M* = 2.99, SD = .58) than when they listened to her voice (*M* = 2.62, SD = .60). A significant main effect of Sexual Orientation, *F*(1, 87) = 7.58, *p* = .007, *η*_*p*_^2^ = .08, was also found. Participants evaluated the heterosexual applicant (*M* = 2. 98, SD = .56) more positively than the lesbian one (*M* = 2.61, SD = .70).^7^

#### Salary

The same ANOVA was conducted, but no significant effects emerged (*F*s < 2.75, *p*s > .10).

#### Traits

We first created two indexes, one for masculine and one for feminine traits (internal reliability ranged from *α* = .81 to *α* = .88). We then performed a 2 (Stimulus: Face vs. Voice) × 2 (Sexual Orientation: Gay vs. Heterosexual) × 2 (Traits: Masculine vs. Feminine) repeated-measures ANOVA with the last variable being a within-subjects factor. A significant interaction between Sexual Orientation and Traits, *F*(1, 88) = 6.42, *p* = .01, *η*_*p*_^2^ = .07, was found. Feminine traits were attributed more to the heterosexual (*M* = 2.68, SD = .49) than to the lesbian applicant (*M* = 2.34, SD = .60; *p* = .004), whereas masculine traits were equally attributed to the lesbian (*M* = 2.56, SD = .61) and to the heterosexual applicant (*M* = 2.36, SD = .65; *p* = .17). Whereas the heterosexual candidate was described with more feminine than masculine traits (*p* = .01), the lesbian candidate was associated equally with feminine and masculine traits (*p* = .21).

#### Correlational Analyses

Correlational analyses were performed between ATL (*α* = .74, overall *M* = 1.41, SD = .44), applicant’s evaluation, salary, and lesbian stereotyping (calculated by subtracting feminine traits from masculine traits). Results did not show any significant correlation between these variables and ATL (*r*s < −.03, *p*s > .21). The only significant correlation emerged between hireability and salary, *r*(92) = .39, *p* < .001. A more likely hireability was related to a higher salary.

#### Indirect Effect

As in Study 2a, the significance of the indirect pathway from applicants’ sexual orientation to hireability via stereotype attribution was assessed using bias-corrected confidence intervals and calculated using 5000 bootstrap resamples (Preacher & Hayes, 2008). This analysis was first conducted regardless of the type of stimulus participants were exposed to, and this is especially because no significant interaction between stimulus and applicant’s sexual orientation was found on candidate’s hireability. The confidence interval of the indirect effect from applicant’s sexual orientation to judgments through the stereotypes included zero (*β* = −.33, SE = .14; CI_95_ = −.003, .214), indicating that overall there was not a significant indirect pathway. Next, the same analysis was conducted for auditory and visual stimuli separately. Whereas in the voice condition, the confidence interval of the indirect effect from applicant’s sexual orientation to hireability through the stereotypes (*β* = −.36, SE = .19; CI_95_ = .033, .37) did not include zero, indicating a significant indirect pathway, this indirect effect did not emerge in the face condition (*β* = −.37, SE = .18; CI_95_ = −.117, .094). Hence, even in this case, it was specifically the voice that induced stereotyping, which in turn affected the candidate’s hireability.

### Gay/Lesbian Categorization or Gender-Atypical Vocal Features?

**Fig. 2:**
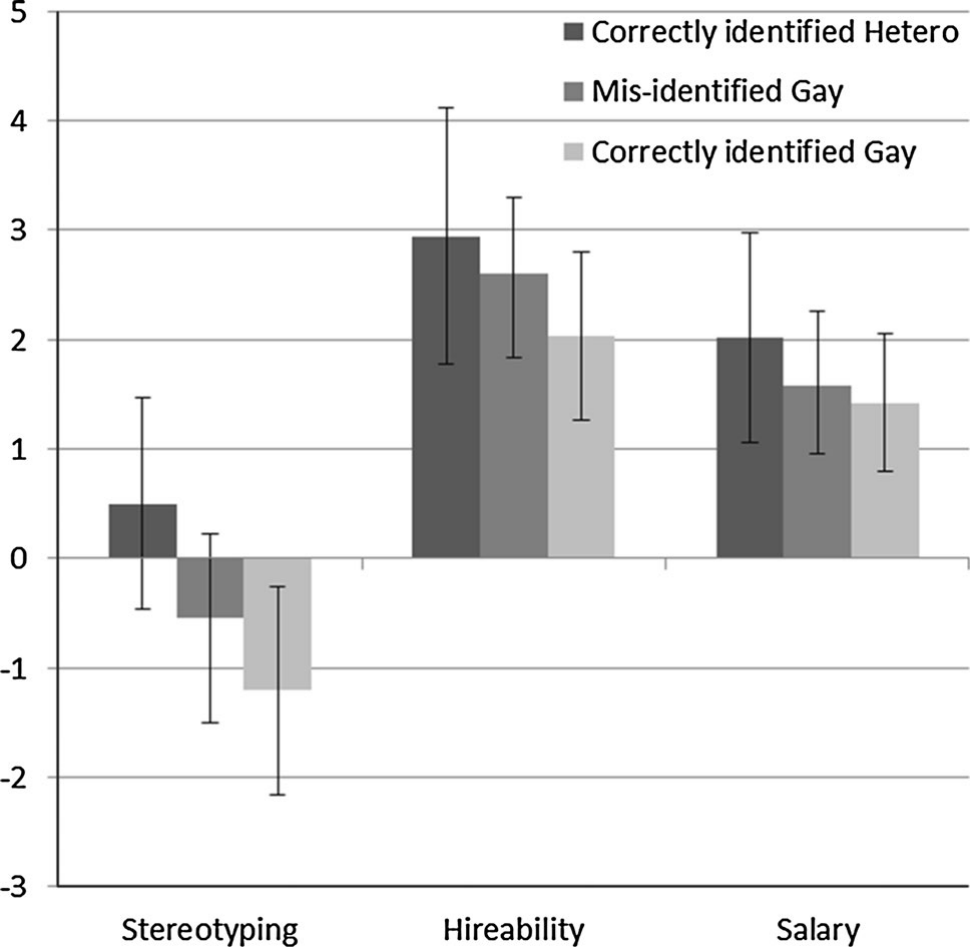
Stereotyping, Hireability, and Salary for correctly categorized heterosexual speakers, misidentified gay/lesbian speakers, and correctly categorized gay/lesbian speakers (Study 2a and 2b)

As in our previous study, we tried to disentangle feature-based from category-based stereotyping and discrimination. We therefore compared the stereotyping and hireability of the (correctly identified) heterosexual speaker with that of gay/lesbian speakers who were or were not correctly identified as gay/lesbian. To simplify, we considered all participants of Study 2. If categorization is a necessary condition for stereotyping and discrimination to occur, only correctly categorized gay/lesbian speakers should be considered as less suitable for the management position than the heterosexual counterpart. However, if a feature-based process is operating, then gay/lesbian speakers should be considered less suitable even when misclassified as heterosexual. A one-way ANOVA was performed for each dependent variable, namely Hireability, Salary, and Gender-Congruent Stereotyping. The latter variable was created by subtracting feminine from masculine traits in the case of male speakers and by subtracting masculine from feminine traits in the case of female speakers. In each case, a reliable main effect emerged for the type of speaker, *F*(2, 139) = 30.64, *p* < .001, *η*_*p*_^2^ = .31, for gender-congruent stereotyping, *F*(2, 140) = 12.33, *p* < .001, *η*_*p*_^2^ = .15 for Hireability, and *F*(2, 140) = 5.87, *p* < .001, *η*_*p*_^2^ = .08 for Salary. In all three cases, means reveal a linear trend, showing greatest stereotyping and discrimination of correctly categorized gay/lesbian speakers, followed by those gay/lesbian speakers that were misidentified as heterosexual (see Fig. 2). Importantly, reliable stereotyping and discrimination, compared to heterosexual speakers, also occurred for gay/lesbian speakers who were misidentified, suggesting that feature-based processes play a role in addition to category-based processes.

## Discussion

Study 2 showed that the men and women who were either perceived as gay/lesbian or simply as deviating from gender norms were rated as inadequate for a leadership position. In the case of male candidates, auditory but not facial features seemed to play a role. Having a heterosexual- rather than a gay-sounding voice created the impression that the speaker had typically masculine traits, which in turn increased the chance to be positively evaluated for the position and to be considered worthy of a higher salary. Indeed, salary is known to be related to perceived masculinity, gender roles, and status (Brescoll, Uhlmann, Moss-Racusin, & Sarnell, 2012).

For female candidates, participants had difficulties in recognizing the candidate’s sexual orientation. Compared with Study 1, this increase in difficulty of judging sexual orientation may be due to the methodology involved (i.e., simultaneous vs. single stimulus presentation). Yet, lesbian candidates were associated with a lack of femininity and hence perceived as gender nonconforming, and received a less positive evaluation than their heterosexual counterparts. Interestingly, even though the type of information (photograph or voice) available to participants mattered less for female applicants, our meditational analyses showed that voice but not face elicited stereotyping, which, in turn, affected evaluation.

These results support the idea of Fassinger et al. (2010) that LGBT leaders need to conform to gender roles to be considered successful. Gay men are not believed to be good leaders, because they lack typically masculine features, whereas lesbian women seem to be a target of discrimination because they deviate from traditional female roles. Also, discrimination does not necessarily require correct recognition of speaker’s sexual orientation; rather, it seemed to be grounded in gender-atypicality and hence may be experienced by any individual (including heterosexuals) lacking in gender-typicality.

## General Discussion

Our findings clearly showed that voice conveys socially relevant information and that listeners drew inferences from vocal cues, even in the case of a private matter such as sexual orientation. These inferences included a broad range of domains (sports, study preferences, and personality) and largely reflected gender-inverted stereotypes such that feminine characteristics were attributed to gay speakers and masculine characteristics to lesbian speakers. This suggests that gender inversion theory (Kite & Deaux, 1987) applies even when listeners form impressions solely on the basis of vocal information contained in limited, single-sentence voice samples.

Importantly, voice not only affected the perception of the speaker as gender-typical/gender-atypical, but also the (intended) behavior toward the speakers. In Study 1a, male (but not female) participants avoided contact with gay individuals although they had only auditory information available, suggesting a subtle impact of voice on social exclusion of gay (gender-atypical) individuals or, vice versa, a preference for heterosexual (gender-typical) in-group members. Study 2 provided further evidence that gay versus heterosexual voice also affected the outcome of a fictitious hiring process. For male job candidates, having a gay-sounding voice created a clear disadvantage when applying for a typically masculine, high-status position (Study 2a). Moreover, women with gender-atypical voices were discriminated when applying for a masculine job, despite the fact that masculinity is generally associated with greater leadership abilities (Schein, Mueller, Lituchy, & Liu, 1996), masculine lesbians are judged as more competent (Niedlich, Steffens, Krause, Settke, & Ebert, 2015), and masculine voice is associated with greater competence (Ko et al., 2009). Hence, heterosexism takes distinct forms for gay and lesbian individuals. This is largely in line with research on backlash demonstrating the gender-specific costs of violating gender-role expectations (Carli & Bukatko, 2000; Rudman & Glick, 2001). In particular, female leaders displaying gender-incongruent behaviors are disliked and sabotaged presumably because they challenge the existing gender hierarchy (Rudman et al., 2012).

If gay/lesbian voice produces both stereotyping and discrimination, as our studies suggest, then one may wonder why this occurs. Are gay/lesbian speakers stereotyped and discriminated because of their sexual orientation or because they do not conform to masculinity/femininity expectations? For instance, are gay men stereotyped and discriminated because they sound gay or because they sound feminine? There is ample evidence that feminine sound (such as high pitch) is associated with reduced competence and leadership capacity both within and across gender groups (e.g., Klofstad et al., 2012; Ko et al., 2009) and that this occurs independently of sexual orientation. Similarly, our data suggest that gender-inverted inferences are common even among those participants who did not identify the speaker as gay or lesbian. For instance, only 9% of lesbian speakers in Study 2b were correctly identified, yet gender-inverted stereotyping was strong. This suggests that gender-atypical voice by itself is sufficient to trigger gender-inverted stereotyping, just as gender-typical voice triggers traditional gender stereotyping. This reasoning is perfectly in line with dual-process models according to which stereotyping may be either feature- or category-driven (Blair et al., 2002). Understanding these different mechanisms is not only of theoretical but also of applied relevance, given that feature-based stereotyping and discrimination are particularly difficult to inhibit (Blair, Judd, & Fallman, 2004). Whereas people may find it relatively easy to avoid discrimination once they have categorized another person as gay or lesbian, they are likely to be unaware of the fact that they are treating another person differently on the basis of their (feminine or masculine) voice. This in turn may make any attempt to control or inhibit discriminatory behaviors in vain.

However, our studies also provided evidence that the categorization of speakers as gay/lesbian versus heterosexual may contribute to stereotyping and discrimination above and beyond the effects of gender-atypical sound. Stereotyping and discrimination tended to be stronger among those participants who correctly recognized the (gay or lesbian) minority status of the speaker when asked to guess sexual orientation. Although additional research is needed on this issue, these findings open the possibility that gay (lesbian) speakers are stereotyped and discriminated on two grounds, namely for sounding feminine (masculine) and for sounding gay (lesbian). Although the two are known to be highly correlated (Smyth et al., 2003; Sulpizio et al., 2015), gender expectancy violation and sexual orientation seem to contribute to stereotyping and discrimination in additive ways.

Given that this research is the first of its kind, it is not surprising that it has a number of limits that ought to be addressed in future studies. First, our speakers were not representative of the general population of heterosexual and gay/lesbian speakers. Rather, we selected from a voice archive of speakers those voices that had a relatively high likelihood of being recognized correctly and that also showed those acoustic characteristics that are usually associated with gay/lesbian and heterosexual speech (Munson et al., 2006; Sulpizio et al., 2015). Thus, we tested the effects of voice on stereotyping and discrimination under somewhat “ideal” conditions, maximizing the chances that voice be revealing of sexual orientation and/or gender (a)typicality. This focus on prototypical rather than on representative samples of voices clearly limits the generalizability of our findings; hence, it remains to be seen whether stereotyping and discrimination are triggered to equal degrees by less prototypical gay voices. Larger and more representative samples, and a direct comparison of speakers of different gender and sexual orientation in a full design, would be needed to draw more stable conclusions. A second limit is that the stereotypical beliefs investigated here may be specific of the Italian context. Whether our findings generalize to other cultures remains a question for future inquiry. Third, we did not investigate the accuracy of the observers’ stereotypical inferences. If gay men and lesbians have gender-atypical interests or traits as some research suggests (e.g., Lippa, 2008) and if observers correctly identify the sexual orientation of the speaker, then the reliance on auditory cues indicative of sexual orientation may indeed lead to (partially) accurate inferences. This seems like a remote possibility to us, given that group stereotypes, even when they contain a “kernel of truth” and correctly describe mean differences between groups, tend to be misleading when applied to single cases because people rarely appreciate the dispersion or variability of traits in a given group (see Judd and Park’s [1993] seminal paper on stereotype accuracy). However, it may be worthwhile for future research to investigate the accuracy of stereotypical inferences based on voice. Unfortunately, we had not obtained any information about our speakers’ actual traits, fields of studies, or preferences, so we cannot draw conclusions about the accuracy of the stereotypical inferences at this point.

Keeping in mind these limitations, our research extends the previous literature in important ways. First, it shows that heterosexual versus gay/lesbian voices are consistently stereotyped in gender-congruent or gender-incongruent ways and that such stereotyping, in turn, leads to differential treatment of heterosexual versus gay/lesbian speakers. To our knowledge, this is the first study showing the unique influence of speakers’ voice conveying sexual orientation on discrimination. Voice-based discrimination on the basis of ethnicity, age, and gender is well established, whereas little is known about how sexual orientation conveyed by voice can affect listeners’ reactions. We found that voice influenced stigmatization and discrimination in subtle ways, affecting not only perception, but also behavior intentions.

Second, by testing both male and female speakers, we have extended prior research that had mostly focused on male speakers. Here we have shown that voice-based stereotyping and discrimination are not a prerogative of gay speakers, but it also affects lesbian speakers. The similarity of stereotyping of male and female speakers is striking, given that the sexual orientation of lesbian women was rarely detected by listeners (for difficult in classifying sexual orientation of women, see Peplau, Spaulding, Conley, & Veniegas, 1999). Also, both gay and lesbian speakers were evaluated more negatively than their heterosexual counterparts when applying for a leadership position, although additional research in actual interview situations (e.g., DeGroot & Motowidlo, 1999) is needed before definite conclusions on voice-based discrimination can be reached.

Third, we extended research on visual cues to auditory information. In the past few years, there has been a growing number of studies investigating how facial features lead to categorization of sexual orientation (Cox et al., 2016; Freeman et al., 2010; Lick & Johnson, 2014; Rule & Ambady, 2008, 2009) and on how facial information influences leader perception (Rule & Ambady, 2011). By comparison, voice remains an under-researched domain. Our findings not only address this lacuna, but they also speak to the differences between auditory and visual information as we directly compared their impact on discrimination in Study 2. Our stimuli were carefully selected so as to be equally telling about the sexual orientation of the target person. In fact, recognition rates were very similar for visual and auditory stimuli, suggesting that participants were equally likely (or unlikely) to detect sexual orientation on the basis of voice or face. Despite this similarity, our findings suggest that inferences drawn from the candidate’s voice were considerably stronger than those drawn from facial features, suggesting that auditory cues may be more informative. This is in line with previous research, showing that, compared to visual cues, auditory cues tend to have a greater impact on social categorization (Rakić, Steffens, & Mummendey, 2011) and on interview judgments (DeGroot & Motowidlo, 1999, Study 2). In our case, the greater impact of voice over face was particularly true for gay male speakers, but was, to some degree, also found for female candidates as the evaluation mediated by stereotyping was driven by auditory and not by visual cues. Hence, it may be that individuals share a well-defined stereotypical idea about how a gay (vs. heterosexual) man sounds, but they may have a more fuzzy idea of lesbian voices (or faces, for that matter). Nevertheless, caution in interpretation is warranted, given the limited number, the type of stimuli used here, and the fact that auditory and visual stimuli were presented separately and belong to different people avoiding us to directly compare information coming simultaneously or from same target. Future research should overcome these stimuli-related limits and addresses the mechanisms driving the potentially more powerful impact of auditory over visual cues related to sexual orientation.

Finally, so far, LGBT leadership has received little attention by experimental research. Our findings suggest that LGBT people who fit the homosexual stereotype are perceived as inadequate for a leadership position. As argued by Fassinger et al. (2010), to be considered a potential leader, LGBT individuals have to endorse typical gender roles, an idea that received empirical support in our study with respect to one specific feature, namely voice. Future research on features such as verbal and nonverbal communication is needed to understand the generality of this “think-manager-think-hetero” phenomenon. Together this research has shown that individuals with gay- or lesbian-sounding voices are at risk of stigmatization and discrimination. The disquieting conclusion is that stigmatization of individuals can easily emerge just from overhearing their voices.

## Footnotes

1 We checked for the acoustic properties of our speakers. For male speakers, compared to the heterosexual speakers, the two gay speakers had a slower speaking rate (*M*_gay_ = 5.49 vs. *M*_hetero_ = 6.66), longer vowel duration (*M*_gay_ = .11 vs. *M*_hetero_ = .08), and a greater/s/center of gravity (*M*_gay_ = 4683.01 vs. *M*_hetero_ = 2778.59). Speakers had a similar pitch: gay (*M* = 104.06, SD = 18.85) and heterosexual speakers (*M* = 100.22, SD = 27.99). Compared to heterosexual speakers, lesbian speakers had a lower F1 and F2 of vowel “a” (F1: *M*_lesbian_ = 6669.55 vs. *M*_hetero_ = 692.47 and F2: *M*_lesbian_ = 1654.63 vs. *M*_hetero_ = 1716.00), “e” (F1: *M*_lesbian_ = 593.34 vs. *M*_hetero_ = 587.48 and F2: *M*_lesbian_ = 1867.67 vs. *M*_hetero_ = 2069.46), and “o” (F1: *M*_lesbian_ = 544.95 vs. *M*_hetero_ = 569.96 and F2: *M*_lesbian_ = 1334.42 vs. *M*_hetero_ = 1618.58). Lesbian (*M* = 172.03, SD = 29.97) and heterosexual (*M* = 207.82, SD = 25.80) speakers had a similar pitch, although the pitch of our lesbian speakers appeared slightly lower. According to what reported by Sulpizio et al. (2015), the acoustic characteristics of these voices make the speakers being perceived as gay/lesbian or heterosexual.

2 Items were selected on the basis of a separate pretest (*N* = 62) in which participants evaluated each sport/field of study as typically masculine/feminine on a scale from 1 (typically masculine) to 7 (typically feminine). The feminine sports (*M* *=* 5.67, SD = .91) were on average perceived as more feminine than the masculine sports (*M* = 1.83, SD = .68; *t*[61] = 20.94, *p* < .001, *d* = 5.36) as well as were the feminine (*M* = 5.66, SD = .68) than the masculine fields of study (*M* = 2.92, SD = .65; *t*[61] = 18.44, *p* < .001, *d* = 4.72). Traits were pretested (*N* = 15) on the same scale. Feminine traits (*M* = 4.53, SD = .39) were perceived as more feminine than masculine ones (*M* = 3.17, SD = .42; *t*[14] = −10.20*, p* < .001, *d* = 5.42).

3 An interaction between Gender and Typicality of Field of Study, *F*(1, 79) = 8.15, *p* = .005, *η*_*p*_^2^ = .09, indicated that men, but not women (*p* = .47), attributed higher likelihood of speakers to be enrolled in feminine (*M* = 2.95, SD = .69) rather than masculine (*M* = 2.52, SD = .67; *p* = .002) field of study.

4 For Sport, a main effect of Gender, *F*(1, 33) = 6.40, *p* = .02, *η*_*p*_^2^ = .16, indicated that female participants (*M* = 2.35, SD = .47) attributed more sports to the speakers than male participants (*M* = 1.96, SD = .46).

5 The two cities mentioned by the male and female speakers are from the same region. As in Study 2b no information about the candidate’ age was provided, we asked participant to rate her age by answering on a scale from 1 (less than 20 years) to 6 (more than 40 years). Differences across condition were found as indicated by the Stimulus by Sexual Orientation interaction, *F*(1, 88) = 6.81*, p* = .01, *η*_*p*_^2^ = .07. While the straight-sounding speakers (*M* = 2.25, SD = .51) were perceived as younger than the lesbian-sounding speakers (*M* = 3.53, SD = 1.72), the candidates portrayed by pictures were similarly rated (*M*_*straight*_ = 3.07, SD = .84 and *M*_lesbian_ = 3.25, SD = .85). However, no relation between perceived age and candidate’s evaluation was found, *r*(92) = .17, *p* = .11. The pattern of results remained the same when perceived age was entered as a covariate in the analyses.

6 Analyses including gender showed no significant main effect or interactions involving this variable for any of the dependent variables.

7 Due to small number of male participants in the sample, gender was not included as factor in the analyses of Study 2b.

## References

[CR1] Ahmed AM, Andersson L, Hammarstedt M (2013). Are gay men and lesbians discriminated against in the hiring process?. Southern Economic Journal.

[CR2] Ambady N, Hallahan M, Conner B (1999). Accuracy of judgments of sexual orientation from thin slices of behavior. Journal of Personality and Social Psychology.

[CR3] Aronovitch, C. D. (1974). *The voice of personality: Stereotyped judgment and their relation to voice quality.* Unpublished doctoral dissertation, Columbia University.

[CR4] Atkins CP, Kent RL (1988). What do recruiters consider important during the employment interview?. Journal of Employment Counseling.

[CR5] Badgett MV, Lau H, Sears B, Ho D (2007). Bias in the workplace: Consistent evidence of sexual orientation and gender identity discrimination.

[CR6] Blair IV, Judd CM, Fallman JL (2004). The automaticity of race and Afrocentric facial features in social judgments. Journal of Personality and Social Psychology.

[CR7] Blair IV, Judd CM, Sadler MS, Jenkins C (2002). The role of Afrocentric features in person perception: Judging by features and categories. Journal of Personality and Social Psychology.

[CR8] Blashill AJ, Powlishta KK (2009). Gay stereotypes: The use of sexual orientation as a cue for gender-related attributes. Sex Roles.

[CR9] Boothroyd LG, Jones BC, Burt DM, Perrett DI (2007). Partner characteristics associated with masculinity, health and maturity in male faces. Personality and Individual Differences.

[CR10] Brescoll VL, Uhlmann EL, Moss-Racusin C, Sarnell L (2012). Masculinity, status, and subordination: Why working for a gender stereotype violator causes men to lose status. Journal of Experimental Social Psychology.

[CR11] Carli BL, Bukatko D, Eckes T, Trautner HM (2000). Gender, communication, and social influence: A developmental perspective. The developmental social psychology of gender.

[CR12] Chemers MM (2000). Leadership research and theory: A functional integration. Group Dynamics.

[CR13] Cox WT, Devine PG, Bischmann AA, Hyde JS (2016). Inferences about sexual orientation: The role of stereotypes, faces, and the gaydar myth. Journal of Sex Research.

[CR14] D’augelli AR, Grossman AH, Starks M (2008). Gender atypicality and sexual orientation development among lesbian, gay, and bisexual youth: Prevalence, sex differences, and parental responses. Journal of Gay & Lesbian Mental Health.

[CR15] DeGroot T, Motowidlo SJ (1999). Why visual and vocal interview cues can affect interviewers’ judgments and predict job performance. Journal of Applied Psychology.

[CR16] Devine PG (1989). Stereotypes and prejudice: Their automatic and controlled components. Journal of Personality and Social Psychology.

[CR17] Dunne MP, Bailey JM, Kirk KM, Martin NG (2000). The subtlety of sex-atypicality. Archives of Sexual Behavior.

[CR18] Eagly AH, Chin JL (2010). Diversity and leadership in a changing world. American Psychologist.

[CR19] Eagly AH, Karau SJ (2002). Role congruity theory of prejudice toward female leaders. Psychological Review.

[CR20] Falomir-Pichastor JM, Mugny G (2009). “I’m not gay…. I’m a real man!”: Heterosexual men’s gender self-esteem and sexual prejudice. Personality and Social Psychology Bulletin.

[CR21] Fasoli F, Maass A, Sulpizio S, Giles H, Maass A (2016). Communication of the “invisible”: The case of sexual orientation. Advances in intergroup communication.

[CR22] Fassinger RE, Shullman SL, Stevenson MR (2010). Toward an affirmative lesbian, gay, bisexual, and transgender leadership paradigm. American Psychologist.

[CR23] Feinberg DR, Jones BC, Law Smith MJ, Moore FR, DeBruine LM, Cornwell RE, Perrett DI, Hillier SG (2006). Menstrual cycle, trait estrogen level, and masculinity preferences in the human voice. Hormones and Behavior.

[CR24] Fink B, Neave N, Seydel H (2007). Male facial appearance signals physical strength to women. American Journal of Human Biology.

[CR25] Freeman JB, Johnson KL, Ambady N, Rule NO (2010). Sexual orientation perception involves gendered facial cues. Personality and Social Psychology Bulletin.

[CR26] Garcia-Retamero R, López-Zafra E (2006). Prejudice against women in male-congenial environments: Perceptions of gender role congruity in leadership. Sex Roles.

[CR27] Gaudio RP (1994). Sounding gay: Pitch properties in the speech of gay and straight men. American Speech.

[CR28] Gowen CW, Britt TW (2006). The interactive effects of homosexual speech and sexual orientation on the stigmatization of men: Evidence for expectancy violation theory. Journal of Language and Social Psychology.

[CR29] Herek GM, Davis CM, Yarber WH, Bauserman R, Schreer G, Davis SL (1998). The attitudes toward lesbians and gay men (ATLG) scale. Sexuality-related measures: A compendium.

[CR30] Herek GM (2000). Sexual prejudice and gender: Do heterosexuals’ attitudes toward lesbians and gay men differ?. Journal of Social Issues.

[CR31] Johnson KL, Lick DJ, Carpinella CM (2015). Emergent research in social vision: An integrated approach to the determinants and consequences of social categorization. Social and Personality Psychology Compass.

[CR32] Jones BC, Feinberg DR, DeBruine LM, Little AC, Vukovic J (2010). A domain-specific opposite-sex bias in human preferences for manipulated voice pitch. Animal Behaviour.

[CR33] Judd CM, Park B (1993). Definition and assessment of accuracy in social stereotypes. Psychological Review.

[CR34] Juodvalkis JL, Grefe BA, Hogue M, Svyantek DJ, DeLamarter W (2003). The effects of job stereotype, applicant gender, and communication style on ratings in screening interviews. International Journal of Organizational Analysis.

[CR35] Kilianski SE (2003). Explaining heterosexual men’s attitudes toward women and gay men: The theory of exclusively masculine identity. Psychology of Men & Masculinity.

[CR36] Kite ME, Deaux K (1987). Gender belief systems: Homosexuality and the implicit inversion theory. Psychology of Women Quarterly.

[CR37] Kite ME, Whitley BE (1996). Sex differences in attitudes toward homosexual persons, behaviors, and civil rights: A meta-analysis. Personality and Social Psychology Bulletin.

[CR38] Klofstad CA, Anderson RC, Peters S (2012). Sounds like a winner: Voice pitch influences perception of leadership capacity in both men and women. Proceedings of the Royal Society B: Biological Sciences.

[CR39] Knöfler T, Imhof M (2007). Does sexual orientation have an impact on nonverbal behavior in interpersonal communication?. Journal of Nonverbal Behavior.

[CR40] Ko SJ, Judd CM, Blair IV (2006). What the voice reveals: Within-and between-category stereotyping on the basis of voice. Personality and Social Psychology Bulletin.

[CR41] Ko SJ, Judd CM, Stapel DA (2009). Stereotyping based on voice in the presence of individuating information: Vocal femininity affects perceived competence but not warmth. Personality and Social Psychology Bulletin.

[CR42] Koenig AM, Eagly AH, Mitchell AA, Ristikari T (2011). Are leader stereotypes masculine? A meta-analysis of three research paradigms. Psychological Bulletin.

[CR43] Lick DJ, Johnson KL (2014). “You can’t tell just by looking!” Beliefs in the diagnosticity of visual cues predict response biases in social categorization. Personality and Social Psychology Bulletin.

[CR44] Lippa RA (2005). Sexual orientation and personality. Annual Review of Sex Research.

[CR45] Lippa RA (2008). Sex differences and sexual orientation differences in personality: Findings from the BBC internet survey. Archives of Sexual Behavior.

[CR46] Locksley A, Hepburn C, Ortiz V (1982). Social stereotypes and judgments of individuals: An instance of the base-rate fallacy. Journal of Experimental Social Psychology.

[CR47] Lyons M, Lynch A, Brewer G, Bruno D (2014). Detection of sexual orientation (“gaydar”) by homosexual and heterosexual women. Archives of Sexual Behavior.

[CR48] McAleer P, Todorov A, Belin P (2014). How do you say ‘hello’? Personality impressions from brief novel voices. PLoS ONE.

[CR49] Munson B (2007). The acoustic correlates of perceived masculinity, perceived femininity, and perceived sexual orientation. Language and Speech.

[CR50] Munson B, McDonald EC, DeBoe NL, White AR (2006). The acoustic and perceptual bases of judgments of women and men’s sexual orientation from read speech. Journal of Phonetics.

[CR51] Niedlich C, Steffens MC, Krause J, Settke E, Ebert ID (2015). Ironic effects of sexual minority group membership: Are lesbians less susceptible to invoking negative female stereotypes than heterosexual women?. Archives of Sexual Behavior.

[CR52] Parnell MK, Lease SH, Green ML (2012). Perceived career barriers for gay, lesbian, and bisexual individuals. Journal of Career Development.

[CR53] Patacchini E, Ragusa G, Zenou Y (2014). Unexplored dimensions of discrimination in Europe: Homosexuality and physical appearance. Journal of Population Economics.

[CR54] Peplau LA, Spaulding LR, Conley TD, Veniegas RC (1999). The development of sexual orientation in women. Annual Review of Sex Research.

[CR55] Petersen T, Togstad T (2006). Getting the offer: Sex discrimination in hiring. Research in Social Stratification and Mobility.

[CR56] Piccoli, V., Carnaghi, A., & Foroni, F. (2015). *TriesteDataBase. Data base on heterosexuals and homosexuals faces*. Unpublished raw data.

[CR57] Powell GN, Butterfield DA, Parent JD (2002). Gender and managerial stereotypes: Have the times changed?. Journal of Management.

[CR58] Preacher KJ, Hayes AF (2008). Asymptotic and resampling strategies for assessing and comparing indirect effects in multiple mediator models. Behavior Research Methods.

[CR59] Puts DA, Hodges CR, Cárdenas RA, Gaulin SJ (2007). Men’s voices as dominance signals: Vocal fundamental and formant frequencies influence dominance attributions among men. Evolution and Human Behavior.

[CR60] Rakić T, Steffens MC, Mummendey A (2011). When it matters how you pronounce it: The influence of regional accents on job interview outcome. British Journal of Psychology.

[CR61] Rieger G, Linsenmeier JA, Gygax L, Garcia S, Bailey JM (2010). Dissecting “gaydar”: Accuracy and the role of masculinity–femininity. Archives of Sexual Behavior.

[CR62] Rojahn K, Willemsen TM (1994). The evaluation of effectiveness and likability of gender-role congruent and gender-role incongruent leaders. Sex Roles.

[CR63] Rudman LA, Glick P (2001). Prescriptive gender stereotypes and backlash toward agentic women. Journal of Social Issues.

[CR64] Rudman LA, Moss-Racusin CA, Phelan JE, Nauts S (2012). Status incongruity and backlash effects: Defending the gender hierarchy motivates prejudice against female leaders. Journal of Experimental Social Psychology.

[CR65] Rule NO (2017). Perceptions of sexual orientation from minimal cues. Archives of Sexual Behavior.

[CR66] Rule NO, Ambady N (2008). Brief exposures: Male sexual orientation is accurately perceived at 50-ms. Journal of Experimental Social Psychology.

[CR67] Rule NO, Ambady N (2009). She’s got the look: Inferences from female chief executive officers’ faces predict their success. Sex Roles.

[CR68] Rule NO, Ambady N (2011). Face and fortune: Inferences of personality from managing partners’ faces predict their firms’ financial success. The Leadership Quarterly.

[CR69] Rule NO, Ishii K, Ambady N (2011). Cross-cultural impressions of leaders’ faces: Consensus and predictive validity. International Journal of Intercultural Relations.

[CR70] Rule NO, Johnson KL, Freeman JB (2016). Evidence for the absence of stimulus quality differences in tests of the accuracy of sexual orientation judgments: A reply to Cox, Devine, Bischmann, and Hyde (2016). Journal of Sex Research.

[CR71] Schein VE, Mueller R, Lituchy T, Liu J (1996). Think manager-think male: A global phenomenon?. Journal of Organizational Behavior.

[CR72] Scherer KR (1978). Personality inference from voice quality: The loud voice of extroversion. European Journal of Social Psychology.

[CR73] Schriesheim CA, Castro SL, Cogliser CC (1999). Leader-member exchange (LMX) research: A comprehensive review of theory, measurement, and data-analytic practices. The Leadership Quarterly.

[CR74] Sczesny S (2003). A closer look beneath the surface: Various facets of the think-manager–think-male stereotype. Sex Roles.

[CR75] Shelp SG (2002). Gaydar: Visual detection of sexual orientation among gay and straight men. Journal of Homosexuality.

[CR76] Smyth R, Jacobs G, Rogers H (2003). Male voices and perceived SO: An experimental and theoretical approach. Language in Society.

[CR77] Snyder K (2006). The G quotient: Why gay executives are excelling as leaders…and what every manager needs to know.

[CR78] Stern C, West TV, Jost JT, Rule NO (2013). The politics of gaydar: Ideological differences in the use of gendered cues in categorizing sexual orientation. Journal of Personality and Social Psychology.

[CR79] Sulpizio S, Fasoli F, Maass A, Paladino MP, Vespignani F, Eyssel F, Bentler D (2015). The sound of voice: Voice-based categorization of speakers’ sexual orientation within and across languages. PLoS ONE.

[CR80] Tigue CC, Borak DJ, O’Connor JJ, Schandl C, Feinberg DR (2012). Voice pitch influences voting behavior. Evolution and Human Behavior.

[CR81] Tilcsik A (2011). Pride and prejudice: Employment discrimination against openly gay men in the United States. American Journal of Sociology.

[CR82] Tskhay KO, Feriozzo MM, Rule NO (2013). Facial features influence the categorization of female sexual orientation. Perception.

[CR83] Tskhay KO, Xu H, Rule NO (2014). Perceptions of leadership success from nonverbal cues communicated by orchestra conductors. The Leadership Quarterly.

[CR84] Valentova JV, Havlíček J (2013). Perceived sexual orientation based on vocal and facial stimuli is linked to self-rated sexual orientation in Czech men. PLoS ONE.

[CR85] Who’s Who: Top 50 OUTstanding in Business List. (October 22, 2013)*. Financial Times*. Retrieved from http://www.ft.com/intl/cms/s/0/476bfcf8-3a15-11e3-9243-00144feab7de.html#axzz3TcB82cJP

[CR86] Zimman L (2010). Female-to-male transsexuals and gay-sounding voices: A pilot study. Colorado Research in Linguistics.

